# Randomized comparison between indocyanine green fluorescence plus ^99m^technetium and ^99m^technetium alone methods for sentinel lymph node biopsy in breast cancer

**DOI:** 10.1038/s41598-019-43473-3

**Published:** 2019-05-06

**Authors:** Charlotte Vermersch, Tiphaine Raia-Barjat, Céline Chapelle, Suzanne Lima, Céline Chauleur

**Affiliations:** 1 Department of Gynecology and Obstetrics, University Hospital, Saint-Etienne, F-42055 France; 2 INSERM U1059, Saint-Etienne, F-42023 France; 3 University of Lyon, Saint-Etienne, F-42023 France; 4 Clinical research, Innovation and Pharmacology unit, University Hospital, Saint-Etienne, F-42055 France

**Keywords:** Health care, Surgical oncology

## Abstract

Use of both patent blue and a radioisotope to locate, and reduce the risk of sentinel lymph node (SLN) detection failure in breast cancer is recommended, but drawbacks commonly lead to using only a radioisotope. An alternative method would therefore be valuable. This randomized, controlled study in 99 patients compared SLN detection using ^99m^technetium (Tc) alone versus Tc combined with indocyanine green (ICG). The primary endpoint was the SLN identification rate. The primary outcome measure was the number of patients with <2 SLN detected. One SLN was detected in 44.0% of patients in the dual detection group and 40.8% in the ^99m^Tc alone group (RR = 1.08 (95% CI 0.68; 1.72), p = 0.84). A mean (±SD) of 2.14 ± 1.23 SLN were identified in the dual detection group *vs*. 1.77 ± 0.85 using Tc alone (*p* = 0.09). Eight-five (78.7%) SLN were both ICG+ and TC+, 15 (13.9%) ICG+ and Tc−, and 7 (6.5%) ICG− and Tc+. SLN detected were ICG-positive in 92.6% of patients and ^99m^Tc-positive in 85.2% with. No adverse event related to ICG injection was recorded. Dual detection of SLN using ICG and radioisotope is reliable and sensitive but was not superior to isotope alone in successfully locating SLN in our pilot randomized trial.

## Introduction

Initial surgery comprising excision of the mammary tumor and sentinel lymph node biopsy (SLNB) is currently the standard of care for early-stage breast cancer^1,2^. The objectives of SLNB are to evaluate extension of the cancer and thereby determine whether complete axillary lymph node dissection is warranted, to improve local control of the disease, and to guide adjuvant medical treatments and postoperative radiotherapy based on a precise staging procedure. Various markers are available for identification of SLN in patients with breast cancer, tracing methods being based on radioactivity, colorimetry or both techniques combined. Each of these three approaches presents specific advantages and drawbacks. The combined (dual) detection method results in a lower rate of false negatives and is consequently still the reference technique^1,3^, enabling closer adherence to the recommendations issued by the French Supreme Health Authority (Haute Autorité de la Santé – HAS) to excise a mean of 2–4 SLN per patient^3^.

The drawbacks of the radioisotope method using radioactive technetium (^99m^Tc), are mainly cost and organizational limitations, the use of patent blue carrying a risk of anaphylactic shock and of persistent tattoos^3,4^. These problems have led numerous teams to abandon the dual detection method, with the consequent risk of an increased rate of false negatives. Development of a new method for SLN identification would be of clinical utility.

The fluorescent tracer indocyanine green (ICG) has been used in clinical practice for the past 40 years^5^. Several feasibility studies have shown it to be a reliable and relevant marker of SLN in patients with breast cancer. ICG could therefore be a valuable alternative marker in this context^6^.

However, few randomized studies have compared the most widely employed technique, featuring the use of a radioisotope alone, to a dual detection method using both a radioisotope and a fluorescent marker^7,8^.

The primary objective of our study was to compare the rates of SLN identification achieved with the dual ICG + 99 mTc detection method and with the 99 mTc method alone. The primary outcome measure was the number of patients with fewer than two (i.e. 0 or 1) SLN detected with the ICG + ^99m^Tc method versus the ^99m^Tc method alone. This primary endpoint, rather than retrieval of at least one SLN (the global definition of detection rate), was chosen in view of our ultimate aim to increase, by use of the dual detection method, the number of SLN retrieved to a mean of 2–4 SLN per patient in accordance with French guidelines.

Secondary objectives were to assess in the dual detection group the proportion of patients with ICG-positive and ^99m^Tc-positive, or ICG-positive and ^99m^Tc-negative, or ICG-negative and ^99m^Tc-positive, or ICG-negative and ^99m^Tc-negative SLN, to determine the time required for SLN biopsy (SLNB) and for surgical intervention, and to report adverse events according to the detection method used.

## Results

### Patient characteristics

A total of 100 patients with clinically lymph node-negative breast cancer were randomized to receive a subareolar injection of either ICG + ^99m^Tc (20 MBq) or ^99m^Tc alone for SLNB. One patient was subsequently excluded, having been enrolled without written consent, resulting in a total of 50 patients in the dual detection group and 49 in the ^99m^Tc alone group. The study was conducted from April 2015 to May 2016. The two groups were comparable in terms of patient characteristics at inclusion, tumor characteristics, rate of invasion of SLN, and per-operative characteristics (Table 1).

**Table 1:** Patient and tumor characteristics.

		Combined detection method (N = 50)	Radioisotope detection method alone (N = 49)	Total N = 99	*P* value
Age (years), mean (SD)		62.2 (12.0)	60.2 (12.5)	61.3 (12.2)	0.42
BMI (kg/m²), mean (SD)		26.8 (4.6)	25.2 (3.9)	26.0 (4.3)	0.08
Post-menopause, no. (%)		34 (68)	30 (62.5)	64 (65.3)	0.67
Tumor diameter (mm), mean (SD)		16.9 (10.8)	20.0 (15.5)	18.4 (13.3)	0.43
SBR grade	I	13 (31)	17 (37.8)	30 (34.5)	0.20
	II	25 (59.5)	19 (42.2)	44 (50.6)	
	III	4 (9.5)	9 (20)	13 (14.9)	
Histological type	IDC	31 (63.3)	33 (67.3)	64 (65.3)	0.58
	ILC	7 (14.3)	4 (8.2%)	11 (11.2)	
	DCIS	6 (12.2)	4 (8.2)	10 (10.2)	
	Other	5 (10.2)	8 (16.3)	13 (13.3)	
Hormone receptor-positive, no. (%)		39 (86.7)	39 (84.8)	78 (85.7)	1.00
HER2 receptor-positive, no. (%)		2 (4.7)	7 (15.2)	9 (10.1)	0.16
Positive sentinel lymph node, no. (%) (micro- or macrometastasis)		18 (30)	17 (34.7)	35 (35.4)	1.00
Complete axillary lymph node dissection for macrometastasis, no. (%)		14 (28)	11 (22.4)	25 (25.3)	0.64

*DCIS* ductal carcinoma *in situ*, *HER2* human epidermal growth factor receptor 2, *IDC* infiltrating ductal carcinoma, *ILC* infiltrating lobular carcinoma, *SBR* Scarff-Bloom-Richardson.

In the dual detection group, fluorescent subcutaneous lymph vessels were visible in 44 patients (88%), allowing the cutaneous incision to be positioned in the axillary fold.

### Primary endpoint

A total of 107 SLN were biopsied in the dual detection group and 87 in the ^99m^Tc alone group. One SLN was detected in 44.0% of patients in the dual detection group and 40.8% in the ^99m^Tc alone group (RR = 1.08 [95% CI 0.68; 1.72]), this difference was not being statistically significant (p = 0.84). More than one SLN were detected in the remaining patients. In all patients at least one SLN was detected, conforming to the global definition of detection rate. The mean number of SLN identified per patient was 2.14 ± 1.23 for the dual detection group and 1.77 ± 0.85 in the ^99m^Tc alone group (p = 0.09) (Table 2).

**Table 2:** Number of sentinel lymph nodes identified.

	Combined detection method (N = 50)	Radioisotope detection method alone (N = 49)	p
**No. (%) of patients with one SLN detected**	22 (44.0)	20 (40.8)	0.84
RR (95% CI)	1.08 (95% CI 0.68; 1.72)		
**Total no. of SLN detected**	108	87	
**No. SLN identified per patient**			
**0**	0 (0.0)	0 (0.0)	
**1**	22 (44.0)	20 (40.8)	
**2**	9 (18.0)	23 (46.9)	
**3**	9 (18.0)	4 (8.2)	
**4**	9 (18.0)	1 (2.0)	
**5**	1 (2.0)	1 (2.0)	
**Mean no. of SLN detected per patient (SD)**	2.14 (1.23)	1.77 (0.85)	0.09
SLN status*			
Negative	84	65	
Micrometastasis	18	12	
macrometastasis	7	10	

*95% CI: 95% confidence interval; RR: relative risk; SLN* sentinel lymph node; * one patient in the combined detection method had micro and macrometastasis.

With regard to sensitivity, SLN were ICG-positive in 92.6% of patients and ^99m^Tc-positive in 85.2%, being both ICG-positive and ^99m^Tc-positive in 78.7% of patients (Table 3). Similar results were found in the 18 patients with lymph node metastasis, SLN were ICG-positive in 91.5% of patients and ^99m^Tc-positive in 80.9% (Table 4).

**Table 3:** Uptake of indocyanine green and ^99m^technetium by sentinel lymph nodes.

	Tc+	Tc−	Total
ICG+, no.(%)	85 (78.7)	15 (13.9)	100 (92.6)
ICG−, no. (%)	7 (6.5)	1 (0.9)	8 (7.4)
Total, no. (%)	92 (85.2)	16 (14.8)	108 (100)

*ICG* indocyanine green, *Tc* technetium.

**Table 4:** Uptake of indocyanine green and ^99m^technetium by metastatic lymph nodes (18 patients with 47 positive sentinel lymph node).

	Tc+	Tc−	Total
ICG+	35 (74.5%)	8 (17%)	43 (91.5%)
ICG−	3 (6.4%)	1 (2.1%)	4 (8.5%)
Total	38 (80.9%)	9 (19.1%)	47 (100%)

### Duration of sentinel lymph node biopsy

SLN excision time was 33.5 ± 17.8 min in the combined detection group and 20.3 ± 11.8 min in the ^99m^Tc detection alone group (p < 0.0001). The overall duration of SLNB was 97.7 ± 39.3 min in the combined detection group *versus* 80.2 ± 34.4 min in the ^99m^Tc group (p = 0.02).

### Adverse events

During the study, 18 (36.0%) patients in the dual detection group and 13 (26.5%) in the ^99m^Tc alone group experienced an adverse event (RR = 1.36 (95% CI 0.75; 2.46), p = 0.39) (Table 5). The two groups did not differ significantly with regard to the incidence of adverse events. No patient experienced an allergic reaction related to ICG.

**Table 5:** Adverse events.

	Combined detection method (N = 50)	Radioisotope detection method alone (N = 49)	p
At least one AE, no. (%)	18 (36.0%)	13 (26.5%)	0.39
RR (95% CI)	1.36 (0.75; 2.46)		
Allergic reactions, no. (%)	0 (0.0)	0 (0.0)	
Seroma, no. (%)	11 (22.0)	6 (12.2)	0.29
Hematoma at the operative site, no. (%)	5 (10.0)	7 (14.3)	0.55
Pain, no. (%)	2 (4.0)	2 (4.1)	1.00

RR: relative risk; CI: confidence interval; no.: number of patients; p: p-value.

## Discussion

Our study was a randomized pilot feasibility trial in close to 100 patients. Unfortunately, the primary endpoint was not reached, only one SLN being detected in 44.0% of patients in the dual detection group and 40.8% in the ^99m^Tc alone group (RR = 1.08 (95% CI 0.68; 1.72). Our goal of a 10% reduction in the percentage of patients with fewer than two SLN detected using the dual detection method was probably too ambitious a target. However, biopsy of a sufficient number of SLN appears to be critical. Excision of too few SLN could increase the rate of false negatives and lead to patients being undertreated^9^. Our objective, by exploring a new detection strategy comprising the dual method (^99m^Tc + ICG) was to assess the value of this new technique for SLN detection and to increase the number of SLN retrieved. The total number of SLNB per patient was higher in the dual detection group than in the radioisotopic detection alone group. In patients whose SLN can be detected, the more SLN identified, the better the tracer. SLN were ICG-positive in 92.6% of patients and both fluorescent and radioactive (i.e. both ICG-positive and ^99m^Tc-positive) in 78.7%. This difference is related to the low molecular weight of ICG, allowing this marker to migrate easily through the lymphatic system, as well as the high sensitivity of fluorescence imaging^9,10^. Numerous publications have reported superiority of this dual detection method^11,12^. In our pilot randomized study, we detected more SLN containing metastases with the dual detection method than with technetium alone (91.5 vs 80.9%). Comparable results were observed in the study of Sugie *et al*. who noted that the additional use of ICG significantly improved detection of metastasis-positive SLN (97.2 vs 90.0%, p < 0.001)^12^. Similarly, Verbeek *et al*. found in two patients SLN containing metastases that had taken up the fluorescent marker, but not the radioisotope^13^.

Use of the dual detection method could lead to a reduced rate of false negatives and enable closer adherence to the recommendations of the French Supreme Health Authority (Haute Autorité de la Santé – HAS) to excise a mean of 2–4 SLN per patient^3^. However, this procedure should not be detrimental to the patient in terms of inducing an increased rate of complications, particularly with regard to lymphedema - a feared complication in patients undergoing complete axillary lymph node dissection. Published reports indicate a significant increase in the rate of lymphoceles and infections following excision of five or more axillary lymph nodes^14^. Our study did not reveal any significant difference in complication rate between the two detection methods evaluated.

Our pilot feasibility study showed a longer duration of SLNB with use of the combined detection. This may be explained by the fact that a gamma probe was not used prior to biopsy of the first fluorescent SLN. Currently, outside the study protocol, we use the two methods alternately, thereby saving time. Use of the ICG marker is particularly valuable when difficulties are encountered, as in the case of patients with a high BMI, or elderly patients in whom SLN detection failures are more frequent. The longer duration of SLNB recorded in the combined detection group may also be explained by the extemporaneous analysis of the SLN excised, using the OSNA method, as the greater the number of SLN analyzed, the longer the time needed to complete the biopsy procedure. As we identified and excised more SLN using the ICG marker, the total time required for their analysis was longer.

In France, use of a dual detection method is recommended by the health authorities (HAS)^3^. In view of the difficulties encountered when combining the use of patent blue with that of a radioisotope, many teams use only a single detection method. Yet even during the initial training period corresponding to the learning curve, the use of a dual detection method could be essential for reducing the rate of false negatives^3^.

As the dual detection method includes a visible component, the fluorescence technique seems to be more rapidly mastered than the radioisotope method, the latter necessitating accomplishment of 30 to 50 procedures for the operator to become fully autonomous^1,3,15,16^. Based on a feasibility study performed in our department, operators can achieve complete proficiency in the fluorescence method after its implementation in 10 patients^17^. Furthermore, this method is easy to implement, the only additional requirements being an ICG solution and a Photodynamic Eye (PDE) or similar camera. ICG is increasingly used for SLN detection in numerous cancers, and is now recommended for SLN detection in certain cancers, such as endometrial cancer. Consequently, many companies have now developed cameras suitable for IGN fluorescence detection and the method has become less expensive.

One of the major advantages of using ICG is the safety of its injection. This results in allergic reactions in fewer than 1/10,000 patients, a substantially lower rate than those reported with the use of patent blue^3,18^. Whereas up to 30% of patients exposed to patent blue experience tattooing of the breast persisting for up to several months, tattooing with ICG, although described, disappears within two weeks to 6 months^19,20^. However, available data are poor and need to be confirmed.

## Conclusion

In this pilot study, we achieved detection of at least one SLN, corresponding to the global definition of SLN identification rate with both detection methods used. However, observed a trend toward a greater mean number of SLNB per patient in the dual detection group, although this difference did not reach statistical significance, and we also detected more SLN containing metastases with the dual detection method than with the use of ^99m^technetium alone. Furthermore, use of this dual detection method was found to be particularly valuable when difficulties in SLN detection were encountered. The Tc99 + ICG dual tracer technique could therefore be of clinical utility, but our preliminary data would need to be confirmed in a larger multicenter study.

## Methods

### Study design and patients

This randomized, open-label, single-center clinical trial compared the combined use of ICG + ^99m^Tc to the conventional radioisotope method using ^99m^Tc alone for SLNB in early-stage breast cancer patients (ClinicalTrials.gov identifier: NCT02279108 Date of registration 30 Oct 2014). The study was approved by the Institutional Review Board (CPP: *Comité de Protection des Personnes No*. *34*–*2014)* in accordance with the Declaration of Helsinki and by the National Commission on Computerization and Freedom (*Commission Nationale de l’Informatique et des Libertés* [CNIL]). Written informed consent was obtained from all patients before their randomization.

Inclusion criteria comprised adult patients with a histologically proved infiltrating breast cancer or a carcinoma *in situ* with an elevated risk of micro-invasion (e.g. a high-grade tumor with a radiologically evaluated diameter over 40 mm, or necessitating immediate mastectomy), unifocal or multifocal but within the same quadrant, with a clinically evaluated diameter <5 cm, absence of palpable axillary lymph nodes or metastasis in preoperative imaging, and no contraindication to radioisotopic detection of SLN.

Patients were randomized in a 1:1 ratio to one of the two study groups using REDCap electronic data capture tools hosted at the University Hospital of Saint-Etienne (France). Randomization was performed using a computer-generated randomization sequence with randomly permuted blocks of 2, 3, 4 or 5.

### Procedures

Patients eligible for SLNB received an injection of 20 MBq of ^99m^Tc- particles in 0.2 mL of saline solution in the subareolar region, either in the afternoon preceding the day of surgery or on the following morning before the operation. Scans of the breast and axilla were performed 2 h post-injection. If no nodes were visualized, a further scan was performed.

For dual detection of SLN, 2 mL of 0.5% ICG solution were injected into the sub-areolar region immediately before surgery. ICG movement along the lymph ducts was facilitated by massage. ICG fluorescence was elicited and detected by a Photodynamic Eye (PDE) camera (Storz, Tägerwilen, Suisse) and lymphatic drainage, revealed by the fluorescent dye, was visualized in real time on a monitor. The camera was fixed to an adjustable articulated arm and positioned at a distance of 20 cm from the surgical field. The fluorescence was traced from the site of injection towards the axilla and an incision was made 1–2 cm proximal to the point where the fluorescence disappeared into the axilla to start the biopsy. Fluorescent (ICG-positive) lymph nodes were then located and excised and the axilla was inspected for any residual fluorescence. Excised ICG-positive nodes were then tested for radioactivity using a gamma probe and classified as hot (^99m^Tc-positive) or cold (^99m^Tc-negative). Finally, the axillary region was checked with the gamma probe to determine whether any radioactivity had been left in place. In the event of significant residual radioactivity, the hot spot (considered to be a ^99m^Tc-positive SLN) was excised and examined.

In each group, the number of SLN (ICG-positive, ^99m^Tc-positive, or both) excised from each patient was recorded. All SLNB were analyzed by the intraoperative one-step nucleic acid amplification (OSNA) method as well as by conventional postoperative histological examination. Patient characteristics, durations of SLNB and surgery, and complications were also recorded.

### Study endpoints

The primary endpoint was the rate of SLN identification achieved with the ICG + ^99m^Tc method versus the ^99m^Tc method alone. The primary outcome measure was the number of patients with fewer than two (i.e. 0 or 1) SLN detected with the ICG + ^99m^Tc method versus the ^99m^Tc method alone. A successful detection rate was defined as retrieval of at least one SLN. The number of SLN detected was determined on the basis of the histopathology report.

The number of SLNB, the time required for SLNB and for the surgical intervention as a whole, and the number and nature of adverse events observed since surgery until the post-operative visit were also compared between the two groups.

### Statistical analysis

All data were collected prospectively and anonymously. Study data were collected and managed using REDCap and Statistical analyses were performed using SAS-Windows^®^ version 9.4 software. The sample size calculation was based on unpublished data obtained in our previous study^21^. The sample size was calculated using MFCalc (http://www.txrating.org/spc/mfcalc/). These data revealed that in 37.5% of patients, fewer than two SLN were identified using the gold standard radioisotope (^99m^Tc) method alone. Our goal was to attain a reduction of 10% in the number of patients with fewer than two SLN identified by use of the dual detection method in order to more closely approach French recommendations to excise a mean of 2–4 SLN per patient. We chose a decrease of 10% in order to match the result achieved with use of patent blue plus radioisotope dual detection^22^. On this basis, we calculated that with a 90% power and a type I error (α) of 5% 45 patients would be needed in each group. However, after incorporating a 5% drop-out rate, we decided to include 50 patients per group. Statistical analyses were performed according to the intention-to-treat principle.

Quantitative variables were presented as mean and standard deviation (SD), qualitative variables being presented as the number and percentages of cases. The rate of detection failures was compared between the dual detection group and the ^99m^Tc alone group using chi-square test. The relative risk and its corresponding 95% confidence interval (95% CI) were also presented. The duration of SLNB and the number of SLN identified were compared between the two groups using the independent-sample *t* test. The threshold of statistical significance was set at *P* < 0.05.

### Ethical approval

All procedures performed in studies involving human participants were in accordance with the ethical standards of the institutional and/or national research committee and with the 1964 Helsinki declaration and its later amendments or comparable ethical standards.

### Informed consent

Informed consent was obtained from all individual participants included in the study.

## Data Availability

The corresponding author, Céline Chauleur, had full access to all data obtained in the study and had final responsibility for the decision to submit the attached manuscript for consideration for publication.
